# A Text-Book of Materia Medica

**Published:** 1885-12

**Authors:** 


### BOOK NOTICES.

A Text-Book of Materia Medica, Characteristic, Analytical, and Comparative. By A. C. Cowperthwaite, M. D., Ph. D., LL. D., Third Edition. Pp. 697; price, $6.00. Chicago: Gross & Delbridge, 1885.

This work has been before the profession for several years, and its character, therefore, must be very well known. For the benefit of those, however, who may not be familiar with it we may say that it is simply an annotated edition of the *Materia Medica.* The symptoms of a drug being stated in the ordinary way, any other remedy having the same or a similar symptom is added in parenthesis. This is an excellent idea for facilitating the study of the materia medica. The work before us had a, to our mind, very humble beginning, but the efforts of the author are evidently appreciated, for this is the third edition, and it is now nearly three times the size of the first issue. Yet much more in the way of notes of comparisons of related remedies might be added with advantage. Thus, under Sulphur we have the symptom, "the child dislikes to be washed and bathed." The similar remedy to this is given—*Ant. crud.* We suggest that the following might be added with propriety:

*Nat. mur.*, desire to wash in cold water. (Hering.)

*Acon.* and *Fl̈uroic acid*, amelioration from washing diseased part. (Lippe.)

*Ant. crud.*, child cries when washed in cold water; better when washed in warm water.

Many other notes might similarly be added throughout the book, increasing its usefulness.

However, as it is it must often prove a welcome assistant in the toil of finding the similar remedy.

We hope that the sale of this third edition will be so rapid that the author may be encouraged to publish another one having many more annotations.

